# NEIGHBORHOOD DISORDER AND COGNITIVE HEATH: THE CAUSAL MEDIATING EFFECTS OF ALLOSTATIC LOAD AND PHYSICAL ACTIVITIES

**DOI:** 10.1093/geroni/igae098.3319

**Published:** 2024-12-31

**Authors:** Jiao Yu, Yi Wang, Xi Chen

**Affiliations:** Yale University, New Haven, Connecticut, United States; Yale University, New Haven, Connecticut, United States; Yale University, New Haven, Connecticut, United States

## Abstract

Numerous studies have attempted to establish a link between neighborhood disorder and later life cognitive decline, with stress accumulation and reduced physical activity hypothesized as potential mediators. However, there remains a significant gap in our understanding of the causal mechanisms underlying this link. This research utilizes data from the Health and Retirement Study (2006-2008 and 2010-2012) to investigate whether stress accumulation, as measured by allostatic load, and physical activity mediate the association between perceived neighborhood disorder and cognitive health. Older Americans aged 65 years and older without dementia at baseline were selected (n=4,346). Cognitive function was measured using the Telephone Interview for Cognitive Status (TICS). Employing propensity score matching followed by causal mediation analyses, we find that higher levels of perceived neighborhood disorder at baseline are associated with poorer cognitive health at a subsequent wave. Under a counterfactual framework, if every respondent had experienced one standard deviation above the mean level of neighborhood disorder, the cognitive score would be 0.68 lower than the score if everyone had experienced one standard deviation below the mean (total effect: -0.68, 95% CI: -0.94, -0.42). Approximately three percent (natural indirect effect = -0.02, 95% CI: -0.04, -0.01) of this effect is attributable to physical activity. Notably, we find no significant mediating effect of allostatic load (natural indirect effect = -0.005, 95% CI: -0.02, 0.004) after adjusting for individual-level characteristics. Our findings underscore the significant role of the behavioral factor in mediating the relationship between disadvantaged neighborhoods and cognitive health among older adults.

